# Supplementary figures and images for: Behavioral determinants for glycemic control among type 2 diabetic patients in Hosanna town; institution based unmatched case control study
Source: PLoS One. 2025 Apr 1;20(4):e0314536. doi: 10.1371/journal.pone.0314536 (PMC11960998; doi:10.1371/journal.pone.0314536)

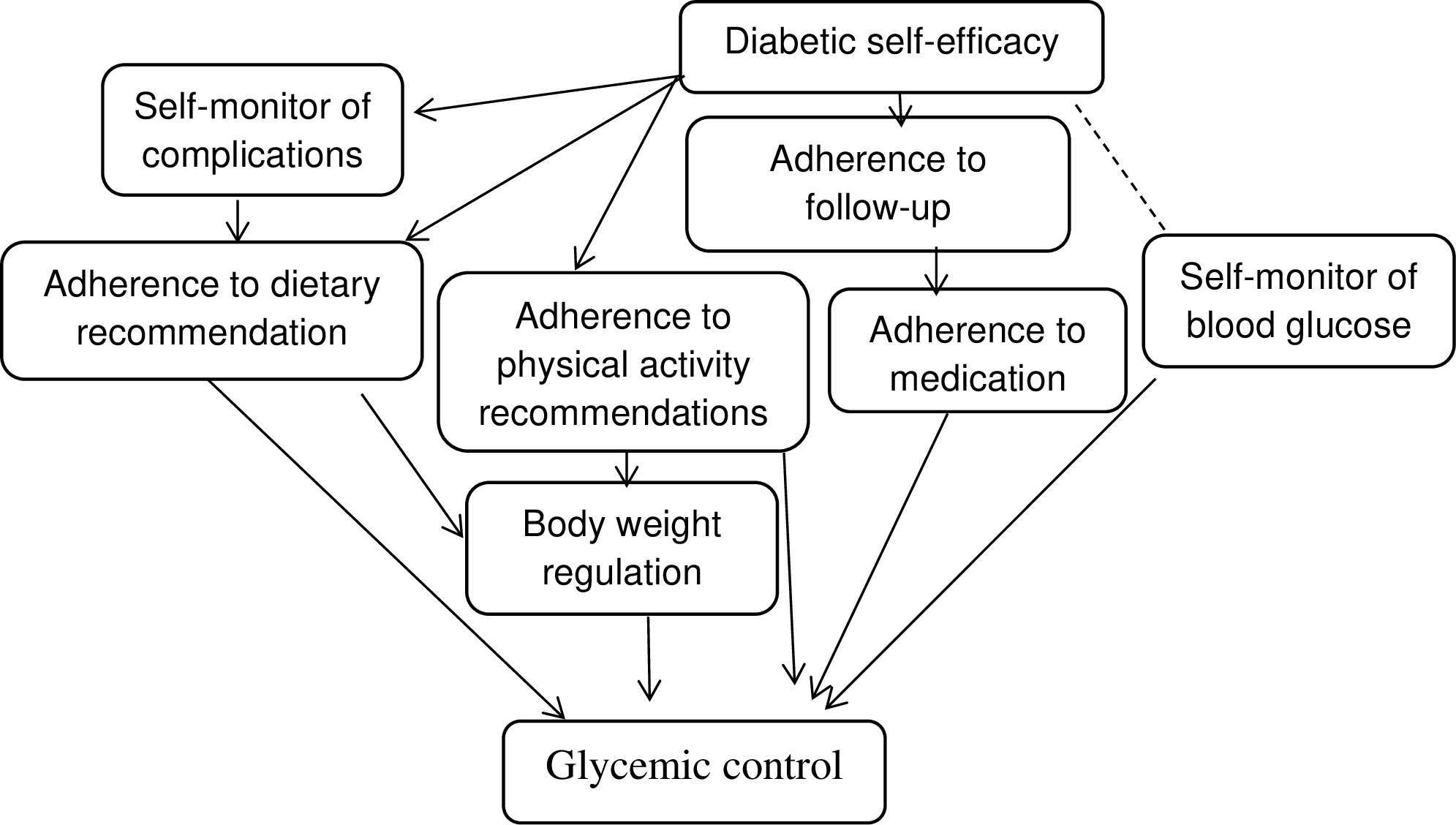

Supplement: S1 Fig — (TIF) [file pone.0314536.s001.tif]

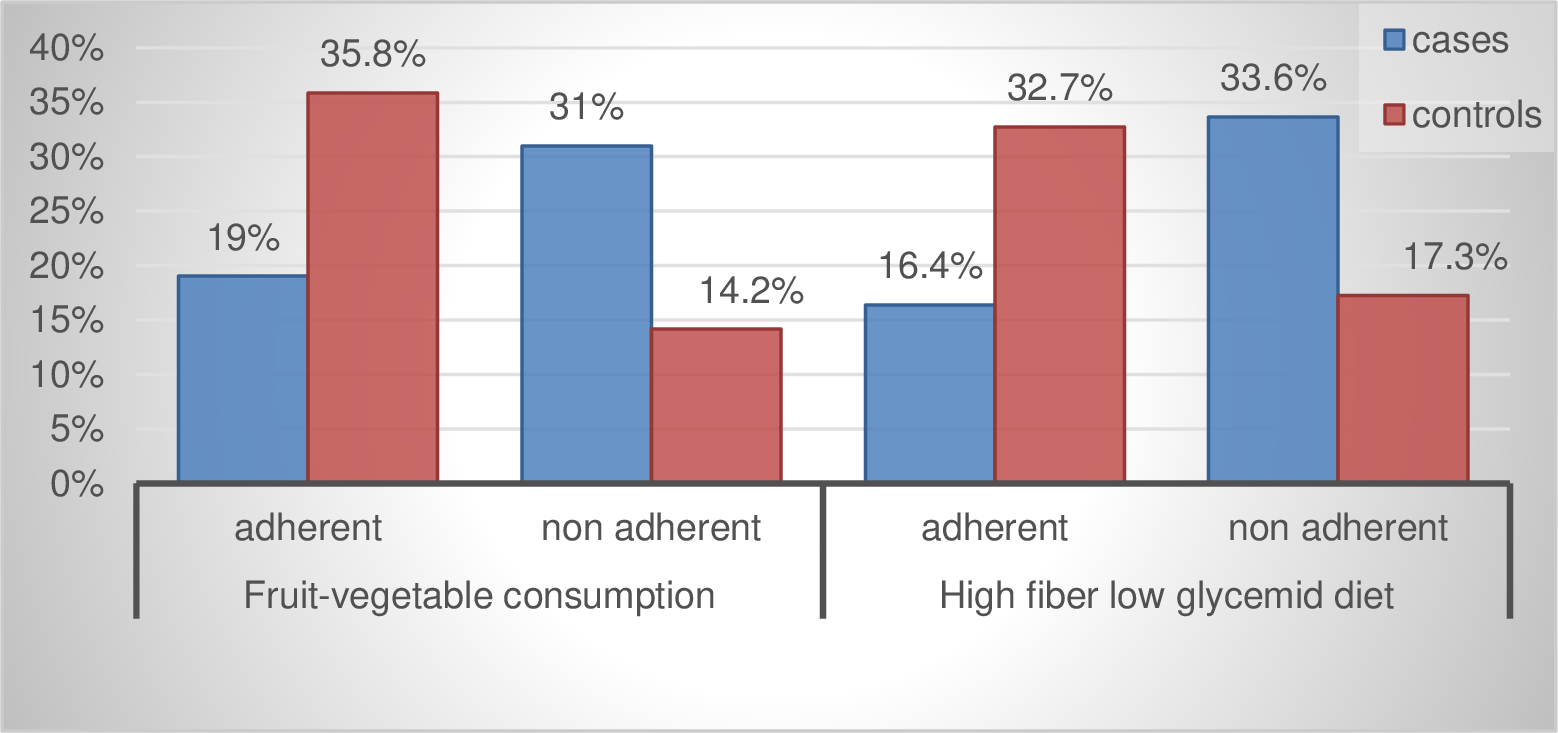

Supplement: S4 Fig — (TIF) [file pone.0314536.s004.tif]
