# Supplementary material for: Behavioral determinants for glycemic control among type 2 diabetic patients in Hosanna town; institution based unmatched case control study
Source: PLoS One. 2025 Apr 1;20(4):e0314536. doi: 10.1371/journal.pone.0314536 (PMC11960998; doi:10.1371/journal.pone.0314536)
Supplement: S2 File — (DOCX) [file pone.0314536.s002.docx]

English version questionnaire

| **Code _______________**  **Interviewer name _______________________________Date ________________ Signature_____________** | | | | | | | |
| --- | --- | --- | --- | --- | --- | --- | --- |
| **Code** | **Part I: Socio-demographic Characteristics** | | | | | | Remark |
| Q101 | Sex of respondent? | 1. Male 2. Female | | | | |  |
| Q102 | Age of respondent **(In year)** | [__________] years | | | | |  |
| Q103 | What is your religion? **(Circle one)** | 1. Orthodox 2. Protestant 3. Muslim 4. Catholic 5. Other(specify) _________ | | | | |  |
| Q104 | Highest level of educational Completed | __________ | | | | |  |
|  | Residence **(Circle one)** | 1. Urban 2. Rural | | | | |  |
| Q105 | Occupation? **(Circle one)** | 1. Government employee 2. NGO 3. Private employed 4. Merchant 5. House wife 6. Farmer 7. Others(specify)___________ | | | | |  |
| Q106 | Your marital status? | 1. Single 2. Married 3. Widowed 4. Divorced | | | | |  |
| Q107 | Your ethnic background? | 1. Hadiya 2. Kambata 3. Amhara 4. Silte 5. Gurage 6. Other (Specify) ____________ | | | | |  |
| Q108 | Duration with Diabetes | __________years | | | | |  |
| **Part II: Diabetes Self Care Behaviors** | | | | | | | |
|  | **Dietary Behavior** | | | | | | |
| Q201 | In a typical week, on how many days do you **eat fruit**? | 1. > 3 days 2. ≤ 3 days | | | | |  |
| Q202 | In a typical week, on how many days do you  **eat vegetables**? | 1. > 3 days 2. ≤ 3 days | | | | |  |
| Q203 | On how many of the last seven days did you eat carbohydrate containing foods with a low Glycemic Index? (e.g., dried beans,  lentils, barley, low-fat dairy products) | 1. > 3 days 2. ≤ 3 days | | | | |  |
| Q204 | On how many of the last seven days did you eat foods high in fiber such as oatmeal, high fiber cereals, and whole-grain bread? | 1. > 3 days 2. ≤ 3 days | | | | |  |
| Q205 | On how many of the last seven days did you space carbohydrates evenly throughout the day? | 1. > 3 days 2. ≤ 3 days | | | | |  |
| Q206 | What type of **oil or fat is most often** used for meal preparation in your household? | 1. Vegetable oil (Liquid 2. Hydrogenated vegetable oil (solid) 3. Table butter (Sheno Lega) 4. Butter or ghee 5. Other (Specify)____________ | | | | |  |
|  | **Alcohol Consumption and cigarette smocking** | | | | | |  |
| Q207 | Have you consumed alcohol [such as beer, wine, tella, tej, areke*]* within the within the **past 7 days**? | 1. Yes 2. No | | | | |  |
| Q208 | Cigarette smoking status | 1. Smoker 2. Non-smoker 3. Ex-smoker | | | | | **IF 2 or 3→Skip to Q210** |
| Q209 | If you smoke, how many cigarettes do you smoke on an average day? | ____________# of cigarettes | | | | |  |
|  | **Physical Exercise:** | | | | | | |
| Q210 | In a typical week, on how many days do you do any vigorous-intensity sports, fitness or recreational (*leisure*) activities that cause large increases in breathing or heart rate like [*running or football,]* ? | 1. 3-7 days/week 2. < 3 days/week 3. Not at all | | | | | **IF 3 →Skip to Q212** |
| Q211 | How much time do you spend doing vigorous-intensity sports, fitness or recreational activities on a typical day? | _________minutes | | | | |  |
|  | **Self-monitoring of blood glucose level** | | | | | | |
| Q212 | Do you’ve access to a device to self-monitor your blood glucose level (SMBG)? | 1. Yes 2. No | | | | | **IF 2→Skip to 216** |
| Q213 | If yes, do you test your blood for sugar? | 1. Yes 2. No | | | | |  |
| Q215 | On how many of the last SEVEN DAYS did you test your blood sugar? | 1. 3-4 times 2. <3 times | | | | |  |
| Q215 | Do you keep a record? | 1. Yes 2. No | | | | |  |
| Q216 | Do you know the normal blood sugar range? | 1. Yes 2. No | | | | | **IF 2→Skip to 218** |
| Q217 | If yes, can you tell me the normal range? | _________Mg/dl | | | | |  |
|  | **Adherence to Medications** | | | | | | |
| Q218 | Compliance to medication in last 7 days | 1. 7 days 2. <7 days | | | | | IF 1**→Skip to 301** |
| Q229 | If < seven days, reason for skipping? | 1. Forget 2. Side effect 3. Other(Specify)____________ | | | | |  |
|  | **Part 3: Self-Efficacy (1=strongly disagree, Disagree,3=not sure, 4=Agree, 5 strongly agree)** | | | | | | |
|  | **I am confident:** |  | | | | | |
| Q301 | I am able to check my blood sugar if necessary | [1] | [2] | [3] | [4] | [5] |  |
| Q302 | I am able to correct my blood sugar when the sugar level is too high | [1] | [2] | [3] | [4] | [5] |  |
| Q303 | I am able to correct my blood sugar when the blood sugar level is too low | [1] | [2] | [3] | [4] | [5] |  |
| Q304 | I am able to choose the correct food | [1] | [2] | [3] | [4] | [5] |  |
| Q305 | I am able to keep my weight under control | [1] | [2] | [3] | [4] | [5] |  |
| Q306 | I am able to examine my feet for cuts | [1] | [2] | [3] | [4] | [5] |  |
| Q307 | I am able to adjust my eating plan when ill | [1] | [2] | [3] | [4] | [5] |  |
| Q308 | I am able to follow a healthy eating pattern most of the time | [1] | [2] | [3] | [4] | [5] |  |
| Q309 | I am able to take more exercise if the doctor advises me to | [1] | [2] | [3] | [4] | [5] |  |
| Q310 | When taking more exercise I am able to adjust my eating plan | [1] | [2] | [3] | [4] | [5] |  |
| Q311 | I am able to follow a healthy eating pattern when I am away from home | [1] | [2] | [3] | [4] | [5] |  |
| Q312 | I am able to follow a healthy eating pattern when I am eating out or at a party | [1] | [2] | [3] | [4] | [5] |  |
| Q313 | I am able to adjust my eating plan when I am feeling stressed or anxious | [1] | [2] | [3] | [4] | [5] |  |
| Q314 | I am able to take my medication as prescribed | [1] | [2] | [3] | [4] | [5] |  |
| Q315 | I am able to adjust my medication when I am ill | [1] | [2] | [3] | [4] | [5] |  |
|  | **Part IV: Check patient card/file and record** | | | | | | |
| Q401 | Random blood sugar level of last three months | __________mg/dl __________ mg/dl  __________ mg/dl | | | | | Mean_______ |
| Q402 | Systolic Blood pressure level (Most recent) |  | | | | |  |
| Q403 | Diastolic Blood pressure level (Most recent) |  | | | | |  |
|  | **Part V: Measurements** | | | | | | |
| Q501 | Height of the patient In meter |  | | | | |  |
| Q502 | Weight of the patient in KG |  | | | | |  |
| Q503 | Waist Circumference |  | | | | |  |
| Q504 | Hip circumference |  | | | | |  |

አማርኛ ትርጉም መጠይቅ

| **ኮድ _______________**  **የመረጃ ሰብሳብው ስም ____________________________ ቀን ________________ ፊርማ_____________** | | | | | | | | | |
| --- | --- | --- | --- | --- | --- | --- | --- | --- | --- |
| **ኮድ** | **ክፍ 1: ማህበራዊና ኢኮኖሚያዊ መግለጫ** | | | | | | | Remark | |
| Q101 | ፆታ? | | 1. ወንድ 2. ሴት | | | | |  | |
| Q102 | ዕድሜ **(በዓመት)** | | [__________] ዓመት | | | | |  | |
| Q103 | ሐይማኖተዎ ምንድ ነው? **(አንድ ብቻ ይክበቡ)** | | 1. ኦረቶዶክስ 2. ፕሮቴስታንት 3. ሙስልም 4. ካቶሊክ   99. ሌላ (ይጥቅሱ) _________ | | | | |  | |
| Q104 | የትምህርት ደረጃ (የመጨረሻዉን ከፍተኛ የደረሱበት) | | __________ | | | | |  | |
| Q105 | የመኖሪያ አድራሻ | | 1. ከተማ 2. ገጠር | | | | |  | |
| Q106 | ሥራዎት ምድ ነው? **(አንድ ብቻ ይክበቡ)** | | 1. መንግስት ሠራተኛ 2. መንግስታዊ ያልሆነ ድርጅት 3. በግል 4. ነጋዴ 5. የቤት እመቤት 6. አርሶ አደር   99. ሌላ (ይጥቅሱ) _________ | | | | |  | |
| Q107 | የጋብቻ ሁኔታ? | | 1. ያላገባ 2. ያገባ 3. ባል የሞተባት/ምስት የሞተበት 4. የተፋታ | | | | |  | |
| Q 108 | ብሔረዎ ምንድ ነው? | | 1. ሃዲያ 2. ካምባታ 3. አማራ 4. ስልጢ 5. ጉራጌ   99.ሌላ (ይጥቅሱ) _________) | | | | |  | |
| Q110 | የስኳር ታማሚ መሆነዎን ካወቁ ምን ያህል ጊዜ ይሆናል | | __________ ዓመት | | | | |  | |
| **ክፍል 2: ለራሰዎ የምያደርጉት እንክብካበ** | | | | | | | | | |
|  | | **የአመጋገብ ሁኔታ** | | | | | | | |
| Q201 | | በሳምንት ውሰጥ ፍራፍሬ ለስንት ቀናት ያክል ይመገባሉ? | 1. > 3 ቀናት 2. ≤ 3 ቀናት | | | | |  | |
| Q202 | | በሳምንት ውሰጥ አታክልት ለስንት ቀናት ያክል ይመገባሉ? | 1. > 3 ቀናት 2. ≤ 3 ቀናት | | | | |  | |
| Q203 | | ከባለፉ ሰባት ቀናት ውስጥ ለስንት ቀን ያክል ዝቅተኛ ካርቦሃይደሬት ያለባቸውን ምግቦችን ለምሳሌ ምስር፣ ባቄላ፣ ገብስ እና በስኳር መጠን አነስተኛ የሆኑ የወተትና ወትት ተዋፅዖ ተመግበዋሉ? | 1. > 3 ቀናት 2. ≤ 3 ቀናት | | | | |  | |
| Q204 | | ከባለፉ ሰባት ቀናት ውስጥ ለስንት ቀን ያክል በፋይበር የበለፀጉ ለምሳሌ አጃ፣ ጥራጠሬ የመሳሰሉትን ተመግበዋሉ? | 1. > 3 ቀናት 2. ≤ 3 ቀናት | | | | |  | |
| Q205 | | ከባለፉ ሰባት ቀናት ውስጥ ለስንት ቀን ያክል ካርቦሃይደሬትን በትክክለኛው ጊዜ ገደብ ወስደው ያውቃሉ? | 1. > 3 ቀናት 2. ≤ 3 ቀናት | | | | |  | |
| Q206 | | ምግብ ለማዘጋጀት ምን ዓይነት የምግብ ዘይት/ቅባት አዘውትረው ይጠቀማሉ? | 1. ዘይት (ፈሳሸ) 2. ዘይት (የሚረጋ) 3. ሸኖ ለጋ (የገበታ ቅቤ) 4. የላም ቅቤ   99.Other (Specify)__________ | | | | |  | |
|  | | **መጠጥ ስለመጠጣትና ስጋ ማጨስ** | | | | | |  | |
| Q207 | | ባለፉት 7 ቀናት አልኮል ያለበት መጠጥ (ቢራ፣ ወይን፣ ጠላ፣ ጠጅ፣ አረቄ) ጠጥቶ ያውቃሉ? | 1. አዎ 2. አይ | | | | |  | |
| Q208 | | ስጋራ አጭሰው የውቃሉ | 1. አዎ 2. አይ 3. አጨስ ነበረ | | | | | **2/3 ከሆነ→ ወደ ጥያቄ Q210 ይለፉ** | |
| Q209 | | ለጥያቄ ቁጥር 209 አዎ ከሆነ በቀን ስንት ስጋራ ያጨሳሉ? | ____________ በቁጥር | | | | |  | |
|  | | **ስፖርት መስራት:** | | | | | | | |
| Q210 | | በሳምንት ለስንት ቀናት ከመካከለኛ እስከ ከፍተኛ የሰውነት እንቅስሰቃሰ የምጠይቅ ስፖርት ለምሳሌ ሩጫ፣ እግር ኳስ መጫወት አተነፋፈስና የልብ ምት መጠን የምጨምሩ ሰርተው ያውቃሉ? | 1. 3-7 ቀናት/በሳምት 2. < 3 ቀናት/በሳምን 3. ምንም አልሰራም | | | | | **3 ከመረጡ → ወደ ጠያቄ ቁጥር Q212 ይለፉ** | |
| Q211 | | እንድህ ያለ ሰፖርት በቀን ለምን ያክል ደቅቃዎች ይሰራሉ? | _________ ደቅቃ | | | | |  | |
|  | | **የራሰዎን የስኳር መጠን መቆጣጠር** | | | | | | | |
| Q212 | | በደመዎ ውስጥ የለውን የስኳር መጠን መለኪያ ለራሰዎ አለዎ? | 1. አዎ 2. አይ | | | | | | **2 ከመረጡ→ወደ ጠያቄ 216 ይለፉ** |
| Q213 | | አዎ ከሆነ ራሰዎ ይለካሉ? | 1. አዎ 2. አይ | | | | | |  |
| Q214 | | ከለኩ ባለፉት ሰባት ቀናት ውስጥ ለስንት ቀናት ያክል የደምዎ ስኳር መጠን ለክተዋሉ? | 1. 3-4 ቀናት 2. <3 ቀናት | | | | | |  |
| Q215 | | የለኩት ስኳር መጠን መዝግበው ስቀምጣሉ? | 1. አዎ 2. አይ | | | | | |  |
| Q216 | | ትክክለኛ የደም ስኳር መጠን ያውቃሉ? | 1. አዎ 2. አይ | | | | | | **2 ከመረጡ→ወደ ጠያቄ 218 ይለፉ** |
| Q217 | | አዎ ከሆነ እስቲንገሩኝ? | ________ሚ.ግ | | | | | |  |
|  | | **የመድኃኒት አወሳሰድ ሰዓት መጠበቅ** | | | | | | | |
| Q218 | | ባለፉት ሰባት ቀናት ውስጥ ለስንት ቀናት ያክል መድኃኒቱን በትክከለኛው ሰዓትና መጠን የወሰዱት? | 1. ለ7 ቀናት 2. <7 ቀናት | | | | | **1 ከመረጡ→ወደ ጠያቄ 301 ይለፉ** | |
| Q219 | | ከ 7 ቀናት ላነሰ ጊዜ ከወሰዱ እንድህ እነድሆኑ ደረገው ምክንያት ምንድ? | 1. መርሳት 2. ጎንዮሽ ጉዳት ስለከበደኝ 3. ሌላ ካለ(ይጥቀሱ)__________ | | | | |  | |
|  | | **ክፍል 3: ያሰቡትን ወይም ያቀዱትን የማከናወን ብቃት/አቅም (1= በጣም አልስማማም, 2= አልስማማም, 3= እርግጠኛ አይደለሁም, 4= እስማማለሁ, 5 = በጣም እስማማለሁ)** | | | | | | | |
|  | | **እርግጠኛ ነኝ:** |  | | | | |  | |
| Q301 | | አሰፈላጊ በሆነ ጊዜ ሁሉ የስኳር መጠነን መለካት እችላለሁ | [1] | [2] | [3] | [4] | [5] |  | |
| Q302 | | የደም ስኳር መጠን በጣም ከፍ ካለ ማስተካከል እችላለሁ | [1] | [2] | [3] | [4] | [5] |  | |
| Q303 | | የደም ስኳር መጠን በጣም ዝቅ ካለ ማስተካከል እችላለሁ | [1] | [2] | [3] | [4] | [5] |  | |
| Q304 | | ለኔ የምሆን ትክክለኛምግብ መምረጥ እችላለሁ | [1] | [2] | [3] | [4] | [5] |  | |
| Q305 | | የራሰን የሰውነት የክብደት መጠንን መቆጣጠር እችላለሁ | [1] | [2] | [3] | [4] | [5] |  | |
| Q306 | | እግሮቼን ያቆሰሉ ነገሮች ካሉ መቆጣጠር እችላለሁI am able to examine my feet for cuts | [1] | [2] | [3] | [4] | [5] |  | |
| Q307 | | ብመኝ እንኳን አመጋገብ ስርኣት ማስተካከል እችላለሁ | [1] | [2] | [3] | [4] | [5] |  | |
| Q308 | | በየትኛውም ጊዜ ጤናማ የአመጋገብ ሂደትን መከተል እችላለሁ | [1] | [2] | [3] | [4] | [5] |  | |
| Q309 | | ሐክም ካዘዘኝ ተጨማሪ ስፖረት እንቅስቃሰዎችን ማከናወን እችላለሁ | [1] | [2] | [3] | [4] | [5] |  | |
| Q310 | | ተጨማሪ ስፖርት የምሰራ ከሆነ አመጋገቡንም ማስተካከል እችላለሁ | [1] | [2] | [3] | [4] | [5] |  | |
| Q311 | | ከቤት ውጭ ስሆን እንኳን የጤናማ አመጋገብ ሂደትን መከተል እችላለሁ | [1] | [2] | [3] | [4] | [5] |  | |
| Q312 | | በተለያዩ ፒሮግራሞችና ግብዛዎች ላይ ብሆን እንኳን የጤናማ አመጋገብ ሂደትን መከተል እችላለሁ | [1] | [2] | [3] | [4] | [5] |  | |
| Q313 | | በውጥረት/ጭንቀት ውስጥ ብሆን እንካን አመጋገብ ስርዓተን ማስተካከል እችላለሁ | [1] | [2] | [3] | [4] | [5] |  | |
| Q314 | | የተዛዘኝን መድኃኒት በአግባቡ መውሰድ እችላለሁ | [1] | [2] | [3] | [4] | [5] |  | |
| Q315 | | ሕመም ብገጠመኝ እንኳን መድኃኒት በአግባቡ መውስድ እችላለሁ | [1] | [2] | [3] | [4] | [5] |  | |
|  | | **ክፍል 4: ከታካሚ ካርድ/መዝገብ የሚሞላ** | | | | | | | |
| Q401 | | ባለፉት ሦስት ወራት የደም የስኳር መጠን | __________ሚ.ግ/ዴ.ሊ __________ ሚ.ግ/ዴ.ሊ  __________ ሚ.ግ/ዴ.ሊ | | | | | አመካይ__ሚ.ግ/ዴ.ሊ | |
| Q402 | | ሲይሶቶሊክ ደም ግፍት መጠን (የቅርብ ጊዜ) | __________ሚ.ግ/ዴ.ሊ | | | | |  | |
| Q403 | | ዲሰቶሊክ ደም ግፍት መጠን (የቅርብ ጊዜ) | __________ሚ.ግ/ዴ.ሊ | | | | |  | |
|  | | **ክፍል 5: እነዚህ ይለኩ** | | | | | | | |
| Q501 | | የታካሚው ቁመት |  | | | | |  | |
| Q502 | | የታካሚ ክብደት |  | | | | |  | |
| Q503 | | የወገብ መጠነ-ዙሪያ |  | | | | |  | |
| Q504 | | የዳሌ መጠነ-ዙሪያ |  | | | | |  | |
