# Supplementary material for: Behavioral determinants for glycemic control among type 2 diabetic patients in Hosanna town; institution based unmatched case control study
Source: PLoS One. 2025 Apr 1;20(4):e0314536. doi: 10.1371/journal.pone.0314536 (PMC11960998; doi:10.1371/journal.pone.0314536)
Supplement: Attachment — Submitted filename: Respnse to Reviewers.docx [file pone.0314536.s006.docx]

***Responses to Reviewers***

Dear editor/reviewers,

We acknowledge the concerns raised by our dear Editor/Reviewers, and we learnt extraordinary lesson during the revision of our manuscript. The following are point-by-point responses to the concerns.

1. Line 59:	Re-written
2. Three months as a reference: We included patients under follow up at least for three months. This is to measure the outcome variable from FBS mean score. In settings where HgbA1c is not feasible for follow-up care, mean FBS used to evaluate the status of glycemic control. We cited this in our case and control identification section
3. Use of FBS over HgbA1c: The hospital does not have the glycated hemoglobin (HbA1c) test for monitoring the glycemic status; instead, FBS is in use as a part of standard of care. We used this data from patients’ record for this study after guaranteeing due ethical clearance and consequently permission.
4. Operational definition
   a. Adherence: Adherence was measured from last seven days status to minimize the risk of recall bias, and this definition was in line with others and we recognized the previous evidences as a reference (cited).
   b. Self-efficacy: it was based on 15 items with 5-point Likert scale and this was cited.
5. We made an extensive revision in the current version to comply with PLOS ONE's style requirements.
6. We sincerely regret any technical error that may have occurred in the submission form with regard to data sharing. Actually we said, *“All relevant data are within the paper and its supporting information files”* under the data sharing sections. The constructed PDF which we downloaded during submission likewise shows this. We shared the data set from our initial submission, and freely shared it in this submission. No restrictions were made on dataset
7. The Ethical statement only appears as a subheading under the Methods section.
8. In response to our esteemed editors, we revised figure 1 and uploaded as a supporting information.
9. We have reviewed our references, and did not come up with retracted papers. Very few of our references are WHO guidelines, Demographic and health survey reports of Ethiopia.
10. The supporting file, S4 SPSS data set.sav (In the current version S4_Data set.sav) is based on IBM SPSS version 23 software. We have re-checked it and faced no difficulty. Even we tried the file from the built pdf link, which was downloaded during submission, it works well. Additionally we shared it again.

| **Reviewers’ concern** | **Response** |
| --- | --- |
| Reviewer #1_Fig 1. | We appreciate our esteemed reviewer;s comment on the issue. However, we recognize an issue as a limitation. |
| Reviewer #1_Fig 2. | Thanks, it was shifted to 2D bar chart. |
| Reviewer #1_Line 229, p value 0.25 | We have described this under the data analysis section of the methodology. Bivariate analysis was done to identify potential variables for multivariable logistic regression. Variables with p-value of <0.25 in bivariate analysis were entered into multivariate analysis. In adjusted model, the level of statistical significance was declared at a p-value of <0.05. Despite that an SPSS output gives us p value of 0, such figures are not real 0. P may approach to 0, but never be 0 in reality. In such cases we used p<0.001. |
| Reviewer #1_Line 230 | The adjustment (multivariable logistic regression analysis) used all variables, which we identified in bivariate analysis. Theses variables were listed in the preceding paragraph. Additionally, we have paraphrased the statement in the revised version. |
| Reviewer #1_Discussion | Concerning issues related to discussion, we were focusing on our objective, behavioral determinant for glycemic control. However if a given factor is either inversely or directly associated with poor glycemic control, we feel that it is also associated with good glycemic control. |
| Reviewer 2_ *“Has this clinical trial been registered on the registration platform?”* | This is an observational study, which we believe that does not require registration. It has gone through necessary ethical review process. We have submitted the ethical clearance and all relevant files pertaining the paper. |
| Reviewer 2_ *“Blood glucose monitoring is also part of the usual care of type 2 diabetes, and whether the data you collect can conduct the analysis of this habit?”* | We have described this in under the section of “Identification of cases and controls” and “Data collection tools and procedures” |

*Sincerely thanks for consideration.*
